# Dietary Thymoquinone Alone or Combined with Swimming Exercise Protect against Microcystin-LR-Induced Oxidative Injury in Mice

**DOI:** 10.1155/2023/5643861

**Published:** 2023-02-22

**Authors:** Ahmed E. Altyar, Amira Hassan Bekhet, Mohamed Kamel, Ghadeer M. Albadrani, Osama A. Kensara, Mohamed M. Abdel-Daim

**Affiliations:** ^1^Department of Pharmacy Practice, Faculty of Pharmacy, King Abdulaziz University, P.O. Box 80260, Jeddah 21589, Saudi Arabia; ^2^Pharmacy Program, Batterjee Medical College, P.O. Box 6231, Jeddah 21442, Saudi Arabia; ^3^Faculty of Physical Therapy, Cairo University, Cairo, Egypt; ^4^Department of Medicine and Infectious Diseases, Faculty of Veterinary Medicine, Cairo University, Giza 12211, Egypt; ^5^Department of Biology, College of Science, Princess Nourah bint Abdulrahman University, P.O. Box 84428, Riyadh 11671, Saudi Arabia; ^6^Department of Clinical Nutrition, Faculty of Applied Medical Sciences, Umm Al-Qura University, P.O. Box 7067, Makkah 21955, Saudi Arabia; ^7^Department of Pharmaceutical Sciences, Pharmacy Program, Batterjee Medical College, P.O. Box 6231, Jeddah 21442, Saudi Arabia; ^8^Pharmacology Department, Faculty of Veterinary Medicine, Suez Canal University, Ismailia 41522, Egypt

## Abstract

Microcystin-leucine-arginine (MCLR) is the most abundant cyanotoxin produced by cyanobacteria. It induces potent cytotoxicity through oxidative stress and DNA damage. Thymoquinone (TQ) is a natural nutraceutical antioxidant derived from black cumin (Nigella sativa). Physical exercise (EX) improves whole-body metabolic homeostasis. Therefore, this study examined the protective role of swimming exercise and TQ against MC-induced toxicity in mice. Fifty-six healthy adult male albino mice (25–30 g) were randomized into seven groups; group (I) was the negative control and received oral physiological saline for 21 days; group (II) received water EX for 30 min daily; group (III) was intraperitoneally injected with TQ (5 mg/kg daily, for 21 days); group (IV) was intraperitoneally administered MC (10 *μ*g/kg daily, for 14 days) and acted as the positive toxic control; group (V) was treated with MC and water EX; group (VI) was injected with MC and TQ; finally, group (VII) was treated with MC with TQ and water EX. In comparison with the control group, the results showed hepatic, renal, and cardiac toxicity in the MCLR-treated group, indicated by a significant increase (*p* < 0.05) in serum levels of alkaline phosphatase (ALP), aspartate aminotransferase (AST), alanine transferase (ALT), cholesterol, lactate dehydrogenase (LDH), creatine kinase (CK), creatine kinase-myocardial band (CK-MB), urea, creatinine, interleukin-6, interleukin -1*β*, and tumor necrosis factor-*α* levels. In addition, there were significant elevations (*p* < 0.05) in malondialdehyde (MDA) and nitric oxide (NO) levels and a significant decrease in reduced glutathione (GSH), glutathione peroxidase (GPx), catalase (CAT), and superoxide dismutase (SOD) in hepatic, cardiac, and renal tissues. Treatment with either TQ or water EX significantly improved (p < 0.05) the MC-induced toxicity with superiority of the TQ group in the restoration of normal ranges; however, cotreatment with both TQ and swimming EX showed the most improvement and restoration to normal ranges as a result of increasing EX clinical efficacy by TQ.

## 1. Introduction

Microcystin (MCs) is a cyclic heptapeptide compound produced by species of *Microcystis* in fresh water and is considered the largest class of cyanotoxins [1]. Exposure to MCs has been proven to result in various adverse health outcomes in animals and humans through cytotoxicity induction. Microcystin-LR (MCLR) is the most toxic variant among the microcystin group. MCLR has a chemically stable formula with multiple routes of exposure, including inhalation, ingestion, or dermal contact with toxin-contaminated waters [2]. One milligram per liter (mg/L) of MC was the recommended limit by the World Health Organization (WHO) in 2020. However, this limit has been exceeded in many aquatic bodies [3]. MC toxicity has grown to be a significant global health issue. Wei et al. reported that MC pollution had been found in various lakes and reservoirs in the waters of 15 Chinese cities. The maximum mean MC concentrations for lakes were reported in Taihu Lake (1.00 *μ*g/L) and Dianchi Lake (23.06 *μ*g/L). For reservoirs, the maximum mean MC concentrations were measured in Yanghe Reservoir (0.98 *μ*g/L) and Guanting Reservoir (4.31 *μ*g/L) [4]. MCLR affects the structure and function of the liver, kidney, brain, and thyroid gland in animals through inhibition of both protein serine/threonine phosphatases-1 and 2A [5–7]; This causes the hyperphosphorylation of proteins, which alters the cytoskeleton causing cell disruption, including cell lysis. Additionally, MCLR induces oxidative stress, which triggers apoptosis, pyroptosis, and tumor promotion [8–11]. Oxidative stress is the imbalance between the synthesis and removal of reactive oxygen species (ROS) that is frequently characterized by alterations in lactate dehydrogenase leakage, lipid peroxidation, and GSH depletion [12].

MCLR exposure at high doses decreases the antioxidant enzyme activities and is accompanied by increased lipid peroxidation. The increased ROS production associated with MCLR toxicity exceeds the capacity of the antioxidant system, which leads to oxidative stress and dysfunction [13]. Antioxidants have a role in decreasing oxidative stress associated with microcystins toxicity. Several studies investigated many antioxidants as protective agents, including piperine, vitamin E, silymarin, or GSH [12, 14, 15]. Moreover, pretreatment with oral antioxidants, including vitamin C or vitamin E, reduced ROS generation and liver injury [16]. However, the exact protection mechanisms from MC-induced toxicity remain questionable. Accordingly, investigating novel antioxidants that offer a high protection level against MC would significantly influence animal and human health.

Thymoquinone (TQ) (2‐isopropyl‐5‐methyl‐1,4‐benzoquinone) is the principal active component (30% to 48%) of *Nigella* sativa (black cumin) seeds essential oil. TQ has therapeutic effects as anti‐inflammatory properties reported in *in-vivo* and *in-vitro* studies [17, 18], in addition to antioxidant properties represented through scavenging ROS and preventing cell damage by various pro-oxidants [19, 20]. TQ's antioxidant properties account for the majority of its health advantages. TQ was reported to decrease oxidative damage in hepatocytes by reducing enzyme activities, including SOD, CAT, and GSH-Px [19, 21, 22].

Regular exercise is a promising nonpharmacological therapeutic strategy impacting mitochondrial metabolism and intracellular signaling processes to enhance renal and hepatic antioxidant activity [23]. Many preclinical and clinical studies have proved the importance of physical activity and exercise training in metabolic improvement. The oxidative stress and inflammatory condition are affected by exercise positively and negatively depending on the exercise type; aerobic (e.g., walking, swimming, cycling, and running) and resistance training are the two main types of exercise [24–28]. Regular aerobic exercise induces adaptations that occur at cellular as well as systemic levels. Aerobic exercise improves the cardiovascular system, significantly lowers ROS, and enhances antioxidant enzyme expression in organs such as the liver, heart, kidney, and brain [24, 25]. Aerobic exercise increases oxygen consumption, affecting the oxidant/antioxidant status [28]. It also helps improve hepatic mitochondrial function, increase fat utilization, and reduce hepatic steatosis [26, 29]. Although exercise's proven antioxidant activity, skeletal muscle is the primary source of ROS during contraction due to the increased metabolic and physical demands associated with exercise. Accordingly, we hypothesize that TQ may improve aerobic exercise's anti-inflammatory and antioxidant efficacy by enhancing its clinical effectiveness and reducing exercise-induced oxidative stress. According to our search, no reports have been published on the effects of TQ and swimming exercises against MCLR toxicity. The current study aimed to evaluate the protective effects of TQ, aerobic swimming exercise, and their combination against MC-oxidative injury in the kidney, liver, and heart tissue.

## 2. Materials and Methods

### 2.1. Chemicals

Microcystin -LR; (CAS No 101043-37-2, molecular weight 995.189 g/mol; purity ≥ 98.5%) and TQ-(2-isopropyl-5-methylbenzo-1,4-quinone, CAS № 490-91-5, molecular weight 2164.204 g/mol; purity ≥ 98.5%) were purchased from Sigma–Aldrich (Saint Louis, MO, USA). Biochemical kits used were procured from the Laboratory Bio Diagnostics Company (Cairo, Egypt), except for lactate dehydrogenase (LDH) kits obtained from (Randox Laboratories Ltd., UK). Interleukin-1*β* (IL-1*β*) and interleukin-6 (IL-6) were purchased from Glory Science Co. Ltd. (Del Rio, TX, USA), and tumor necrosis factor-*α* (TNF-*α*) was obtained from Bio Source International Inc. (Camarillo, CA, USA) for inflammatory reaction assessment.

### 2.2. Animals

Fifty-six healthy male albino mice, weighing 25–30 g, were kept in wire-mesh metallic cages and housed using controlled conditions, temperature (25±2°C) with 12 h light/ dark cycle. They were acclimatized to the surrounding conditions for seven days before the study, and water and food were served ad libitum. The Institutional Animal Care and Use Committee, Faculty of Science, Cairo University, Egypt, revised and approved the study protocols and mice investigations. All experimental procedures, sampling methods, animal dealing, and scarification occurred according to international guidelines for the use and care of experimental animals.

### 2.3. Experimental Groups and Design

Animals were equally distributed into seven experimental groups (*n* = 8/group).

Group I(control): animals were given oral physiological saline. Group II: animals (water exercise group) [27] training program consisted of swimming for 30 min daily in (30 x30 × 40 cm) filled tank using warm water with 25 cm depth to inhibit the mice from supporting themselves using tails touching the bottom of the tank. Group III (TQ group): animals were given thymoquinone (5 mg/kg BW/day for 21 days i.p; Talib (2017)) [30]. Group IV (MCLR group): animals received microcystin(10 *μ*g/kg BW/day, i.p) [31] for 14 days, followed by distilled water for the rest of seven days. Group V (MCIR-TQ): animals were given TQ alone for seven days, followed by TQ and MCIR *s*imultaneously for the rest of 14 days. Group VI (MCIR-swimming Ex): animals trained with the same swimming exercise regimen for seven days, followed by MCLR and swimming exercise for the rest of 14 days. Group VII (MCIR-TQ-swimming EX): animals had TQ and exercise program for seven days, then received MCIR-TQ and swimming exercise for the remaining 14 days.

### 2.4. Blood Sampling and Tissue Preparation

Twenty-four hours after the last treatment, the animals of all groups were euthanized using an overdose of sodium pentobarbital then blood samples were gathered through heart puncture. The collected samples were left for clotting, then centrifuged at 1200 g for 10 min and stored at −20°C until the assessment of the biochemical parameters. Hearts, kidneys, and livers were excised and homogenized using ice-cold 0.2 M Tris-HCl buffer, pH 7.4, followed by refrigerated centrifugation at 10,000 × g. (4°C), the resulting supernatant was collected and stored at −80°C to determine oxidative cascade markers within the tissue.

### 2.5. Serum Biochemical Assay

Serum hepatic and renal specific markers were estimated as aspartate transferase (AST), serum alanine transferase (ALT) using Reitman and Frankel [32] method, alkaline phosphatase (ALP) was measured using the technique of Tietz et al. [33], while serum cholesterol and lactate dehydrogenase (LDH) levels were estimated using the method of Babson and Babson; Allain et al. [34, 35]. Moreover, urea and creatinine levels were calculated using the techniques described by Coulombe and Favreau; Larsen [36, 37], respectively. Proinflammatory cytokines, IL-1*β*, IL-6, and TNF-*α*, were evaluated using a commercially available ELISAs Kit according to the manufacturer's instructions; absorbance values are determined using an automated ELISA reader at 450 nm. Creatine Kinase (CK) was evaluated according to the method developed by Szasz et al. [38]. While CK-MB was measured according to Wurzburg et al. [39].

### 2.6. Lipid Peroxidation and Antioxidant Assays

Assessment of lipid peroxidation biomarker malondialdehyde (MDA) was made in hepatic, renal, and cardiac tissue according to Uchiyama and Mihara [40] and nitric oxide (NO) concentration according to the method of Green et al. [41] to indicate the oxidant/antioxidant status in addition to measuring the tissue level of GSH using Beutler et al. [42] method, glutathione peroxidase (GSH-PX) using Paglia and Valentine [43], catalase (CAT) using Aebi [44], and superoxide dismutase (SOD) using Nishikimi et al. method [45].

### 2.7. Data Analysis

Data were statistically reported in terms of mean and standard deviation (SD). The normality of data was checked using Shapiro–Wilk's test and for homogeneity using Levene's test, then, compared using the one-way analysis of variance (ANOVA) test with Tukey's test for the significance of difference evaluation between means. *p* values less than 0.05 was considered statistically significant. The computer program IBM SPSS Statistical Package for the Social Science was used in all statistical calculations; IBM Corp, Armonk, NY, USA version 22 for Microsoft Windows.

## 3. Results

### 3.1. Serum Biochemical Analysis

**Figure 1:**
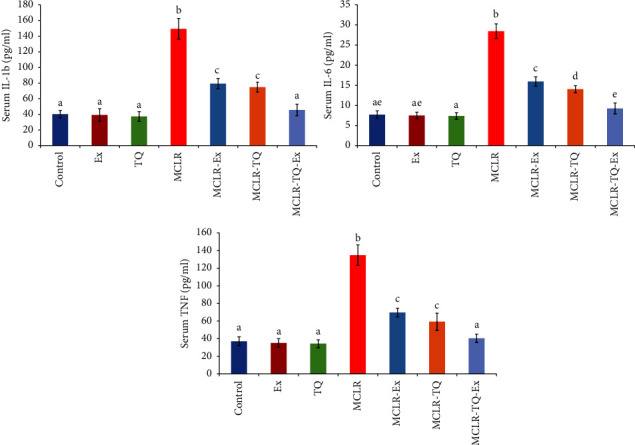
The biochemical effects of swimming exercise and thymoquinone (5 mg/kg BW) against microcystin-LR (10 *μ*g/kg BW/day) on inflammatory markers, Columns (means ± SD) with different superscripts show significant differences (*p* < 0.05) between groups.

Compared to the control group, the MCLR-intoxicated group presented significant elevation (*p* < 0.05) in serum concentrations of hepatic ALT (143%), AST (119%), ALP (125%), renal urea (136%), and creatinine biomarkers (1236%), in addition to elevation of serum LDH (70%), cholesterol (130%), CK (178%), CK-MB (313%) (Table 1), IL-1*β* (271%), IL-6 (268%), and TNF-*α* (by 265%) (Figure 1).

MCLR-intoxicated group with TQ, swimming exercise, or both presented improvement in all serum biochemical changes by presenting a significant decrease in ALT by 35%, 32%, and 54.9%, respectively; AST by 41%, 22%, and 48%, respectively; ALP by 37%,30%, and 52%, respectively; renal urea by 41%,28%, and 54%, respectively; and creatinine biomarkers by 58%, 48%, and 85%, respectively, in addition to elevation of serum LDH by 24.6%, 21.3%, and 37.9%, respectively; cholesterol by 32.6%, 24.6%, and 53.8%, respectively; CK by 44%,40%, and 60, respectively; CK-MB by 51%, 45%, and 73%, respectively; (Table 1), IL-1*β* by 50%, 47%, and 69%, respectively; IL-6 (by 51%, 44%, and 67%, respectively; and TNF-*α* by 56%, 48%, and 70%, respectively, MCLR-treated group with TQ showed more improvement compared to MCLR-treated group with exercise. Treatment of the MCLR-intoxicated group with TQ and swimming exercise had more restoration of normal control ranges with an insignificant difference(*p* > 0.05) with the control group in all serum biochemical changes.

### 3.2. Antioxidant Activity in the Hepatic Tissue

**Figure 2:**
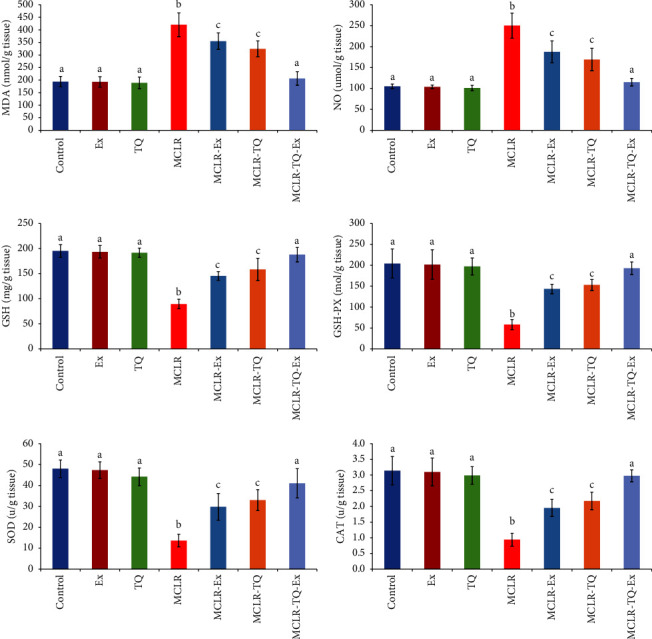
The protective effects of swimming exercise and thymoquinone (5 mg/kg BW) against microcystin-LR (10 *μ*g/kg BW/day) on hepatic tissue MDA malondialdehyde, NO nitric oxide, GSH reduced glutathione, GSH-PX glutathione peroxidase, and SOD superoxide dismutase, CAT catalase. Columns (means ± SD) with different superscripts show significant differences (*p* < 0.05) between groups.

Compared to the control group, the MCLR-intoxicated group exhibited a significant decrease (*p* < 0.05) in hepatic tissue concentrations of GSH by 54%, GSH-PX by 72%, SOD by 72%, and CAT by 70%. Moreover, MDA and NO concentrations in the hepatic tissue were significantly increased (*p* < 0.05) after MCLR- intoxication (by 117% and 138%), respectively. Treatment of MCLR-intoxicated group by TQ, swimming exercise, or both was associated with a significant decrease in MDA by 22.9%, 15.5%, and 50.9%, respectively, and NO by 32%, 25%, and 54%, respectively; when compared to the MCLR group. While there is a significant increase observed in the MCLR-treated groups by TQ, swimming exercise, or both in GSH concentration by 77%, 62.6%, and 110%, respectively, in addition to the antioxidant enzymes activities (GSH-PX by 162%, 146.6%, and 321%, SOD by 141%, 118%, and 200%, and CAT by 130%, 107%, and 215%, respectively) compared to MCLR-treated group, improving these parameters to the normal ranges (Figure 2).

### 3.3. Antioxidant Activity in the Renal Tissue

**Figure 3:**
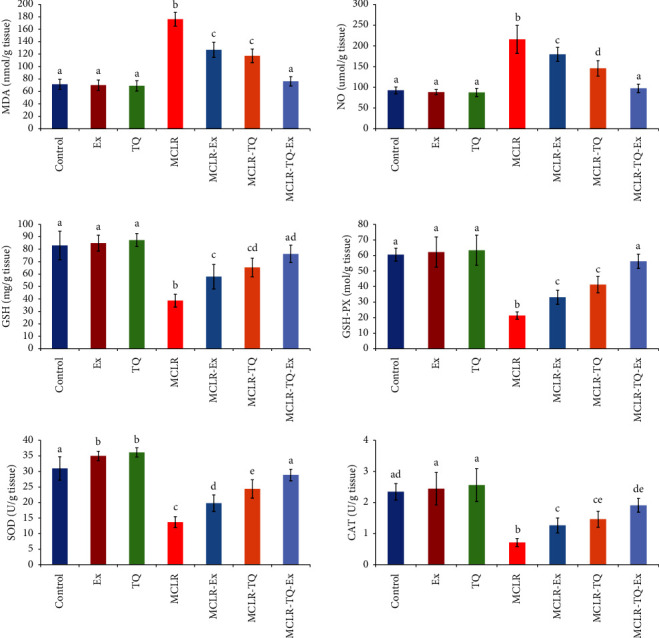
The protective effects of swimming exercise and thymoquinone (5 mg/kg BW) against microcystin-LR (10 *μ*g/kg BW/day) on renal tissue MDA malondialdehyde, NO nitric oxide, GSH reduced glutathione, GSH-PX glutathione peroxidase, SOD superoxide dismutase, and CAT catalase. Columns (means ± SD) with different superscripts show significant differences (*p* < 0.05) between groups.

The MCLR-intoxicated group expressed a significant increase (p < 0.05) in renal tissue MDA and NO concentrations (by 147% and 133%, respectively) compared to the normal group. At the same time, there were significant drops in renal tissue GSH concentrations and activities of GSH-PX, SOD, and CAT by 53%, 65%, 56%, and 70%, respectively, compared to the control group. Treatment of MCLR-intoxicated groups with TQ or swimming exercise significantly reduced MDA and NO concentrations (MCLR-TQ: 33.5%, 32.4% and MCLR-swimming exercise: 27.9%, and 16.18%, respectively) and increased GSH concentrations and activities of GSH-PX, SOD, and CAT (MCLR-TQ: 69%, 93%, 78%, 105% and MCLR-swimming exercise: 49.8%, 55%, 44.6%, and 77%, respectively) in comparison to MCLR group. A combination of TQ and swimming exercise expressed more improvement in the renal antioxidant status of MCLR-intoxicated groups, where there was a reduction in MDA and NO by 56.7% and 54.8%, respectively, and elevations in GSH, GSH-PX, SOD, and CAT by 97%, 163%, 111%, and 169%, respectively, when compared to the MCLR group (Figure 3).

### 3.4. Antioxidant Activity in the Cardiac Tissue

**Figure 4:**
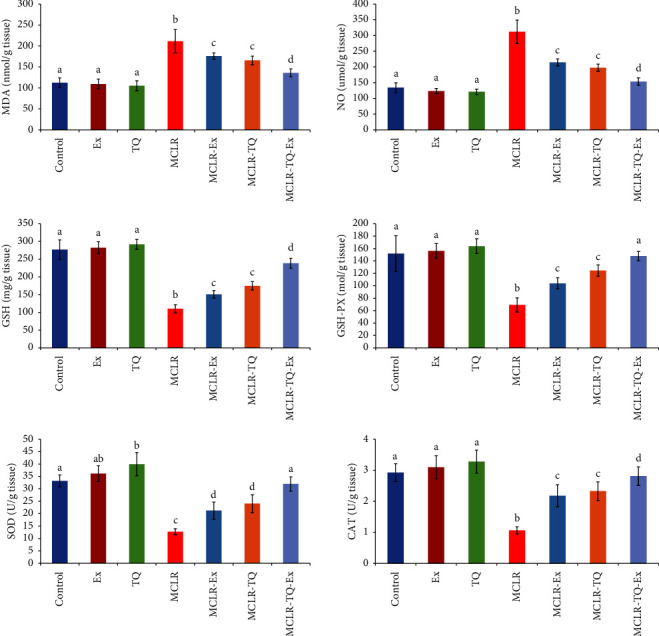
The protective effects of swimming exercise and thymoquinone (5 mg/kg BW) against microcystin-LR (10 *μ*g/kg BW/day) on cardiac tissue MDA malondialdehyde, NO nitric oxide, GSH reduced glutathione, GSH-PX glutathione peroxidase, SOD superoxide dismutase, and CAT catalase. Columns (means ± SD) with different superscripts show significant differences (*p* < 0.05) between groups.

Microcystin intoxication triggers oxidative damage in the heart, expressed in a significant elevation (*p* < 0.05) in MDA and NO concentrations by 88% and 132%, respectively, and drops in GSH, GSH-PX, SOD, and CAT by 60%, 54%, 62%, and 64%, respectively, in comparison to the normal group. Moreover, treatment of MCLR- intoxicated group with TQ or swimming exercise improved the MC-LR- induced cardiac oxidative damage by significant reductions (*p* < 0.05) in MDA by 21.9%, and 16.7%, respectively, and NO by 36.6%, and 31.26%, respectively, and elevation in GSH levels, GSH-PX, SOD, and CAT (MCLR-TQ: 59%, 79.8%, 88%, and 119.8%, respectively, and MCLR-swimming exercise: 36.9%, 50%, 66%, and 105.6%, respectively) compared to MCLR- intoxicated rats. The treatment combination of TQ and swimming exercise improved the induced cardiac oxidative stress more than every single treatment, expressed by decreasing cardiac MDA and NO (by 35.7% and 50.7%) and increasing cardiac GSH, GSH-PX, SOD, and CAT by 157.7%, 113.4%, 150%, and 165%, respectively compared to the MCLR-intoxicated group (Figure 4).

## 4. Discussion

In the current study, we examined the anti-inflammatory and antioxidant properties of TQ alone or combined with swimming exercise against MCLR-induced oxidative damage in hepatic, renal, and cardiac tissues. Our data presented MC-induced hepatotoxicity, nephrotoxicity, and cardiotoxicity indicated by significantly elevated serum AST, ALT, ALP, LDH, CK, and CK-MB enzyme activities. Moreover, cholesterol, urea, and creatinine were elevated. In addition to oxidative stress, significant increases in MDA and NO levels are observed with the depletion of cellular antioxidants in the liver, kidney, and heart tissues.

The microcystins are the most abundant toxins found in fresh water, causing hepatorenal injuries as potent acute hepatotoxicity due to inhibition of serine/threonine protein phosphatases (PPs) as PP1 and PP2A 1 through strong covalent bond formation leading to excessive phosphorylation to many cellular proteins, cytoskeleton alterations and loss of integrity. Significant negative impact on cell homeostasis resulted from uncontrolled PPs inhibition and kinases hindering the balance of protein phosphorylation/dephosphorylation, leading to overflow of liver marker enzymes into blood and elevation of ALT, AST, and ALP levels [12, 46, 47]. Our results were in accordance with the data of previous studies [15, 46, 48].

Despite the robust defense system, an increase in the formation of ROS or a reduction in the antioxidant capacity can result in gradual cell damage and a deterioration in physiological performance. The homeostatic balance is interrupted, in addition to shifting the redox state toward more pro-oxidizing when the oxidant capacity exceeds the antioxidant capacity.

The main function of antioxidant defensive systems is to counter the effect of reactive species through nonenzymatic and enzymatic addition to dietary antioxidants. Glutathione, uric acid, bilirubin, coenzyme Q10, and lipoic acid are nonenzymatic antioxidant agents that originate from endogenous sources and are frequently by-products of cellular metabolism. At the same time, the main enzymatic antioxidants are glutathione peroxidase (GPX), superoxide dismutase (SOD), and catalase.

Similar to the findings of Lowe et al. [48], there is a significant deterioration in renal physiological parameters expressed as elevation of urea and creatinine in the MCLR-treated group; they reported an increase in glomerular filtration rate, proteinuria, renal index, and sodium excretion in addition to the structural changes in renal tissue. Several studies have linked MC-induced hepatotoxicity to the high affinity of MCLR to form strong covalent bonds with hepatic serine/threonine-specific PPs leading to their inhibition. In addition, we observed a significant increase in serum cholesterol and LDH levels, which may be due to the MCLR-induced hepatic injury.

Inhibition of protein phosphatases caused by MCLR leads to overphosphorylation of many proteins associated with hepatic, renal, and cardiac oxidative injuries. It raises the intracellular lipid peroxidation, producing the oxidation products that represent the cornerstone of oxidative signaling. MC-induced oxidative stress is expressed as an increase in the production of ROS, such as O2•−, H2O2, and OH•. This overproduction indicates a disturbance in the body's normal redox state, leading to cellular lipids injury, ATP reduction, DNA oxidative damage, and mitochondrial dysfunction [46, 47].

By the findings of Abdel-Daim et al. [15], we observed a significant elevation in MDA and NO levels and a significant reduction in antioxidant enzymes, such as GSH, GPx, SOD, and CAT, acting as defense mechanisms through hydrolysis of H_2_O_2_ into H_2_O. The depletion of antioxidant enzymes could be explained by overutilization in overcoming free radicals produced during MC metabolism.

The present study reported a significant elevation in IL-1*β*, IL-6, and TNF- *α*. These findings were in agreement with Cao et al., who noted that MCLR low dose concentration stimulates the production of the proinflammatory factors mRNA of TNF-*α*, IL-1*β*, and IL-6 [49]. However, as a result of a high dose, in addition to the prolonged stress of MCLR, the mice's immune systems are severely damaged, and cell secretion of inflammatory factors deteriorates as a result of its apoptosis or necrosis [50].

Our data revealed that TQ and exercise reduce oxidative injuries and improve biochemical alterations induced by MCLR. TQ action may be an antioxidant agent that prevents the peroxidation of membrane lipids in hepatic cells through acting as general free radical scavengers, ROS due to MC toxicity, attacks cellular membrane lipids resulting from lipid peroxidation leading to MDA level elevation as the final product of lipid peroxidation. It acts as an index indicating lipid peroxidation. In our study, the height of MDA in hepatic, renal, and cardiac tissues has ameliorated after the mice's exposure to TQ and exercise.

The effects of one bout of exercise and regular physical activity are dissimilar in cell adaptation to the elevated ROS production to be more resistant to the negative impact of oxidative stress. Regular physical activity has various advantages, and the body adapts to the increased oxidant levels; conversely, acute exercise increases only a minimal amount of adaptation [23, 24, 51]. Although skeletal muscle is relatively resistant to exercise-induced oxidative damage, it is clear that prolonged and/or intense exercise has negative effects. Antioxidant supplementation is widely used with high levels of physical activity. It has a role in preventing exercise-induced oxidative damage [52].

Several studies support a positive association between regular aerobic exercise and decreasing oxidative stress [24, 26, 53]. It has been reported that regular aerobic exercise causes an increase in maximal oxygen consumption (VO2max) and increases ROS production. However, if aerobic exercise intensities do not exceed 50% of VO2max, ROS production is decreased to the minimum values, as demonstrated in studies by Ashton et al. [54] or Chevron et al. [55]. These findings indicate that TQ and exercise are effective in lipid peroxidation prevention and the anti-inflammatory and immunomodulatory effects of TQ [56–58]. Our results are in accordance with the conclusions of previous reports that investigated the antioxidant and anti-inflammatory effects of TQ [15, 59]. In our study, the swimming exercise was effective against MC-induced metabolic changes. Exercise suppresses endotoxin-induced TNF-a through normalizing overexpression of TNF-a, and the exercise's anti-inflammatory effects protect against chronic systemic low-grade inflammation induced by toxicity [53, 60]. Our data are in accordance with those presented by Booth et al. [61], they showed beneficial results of physical exercise on the liver and kidneys.

Swimming exercise was associated with significant amelioration of MCLR-induced elevation of AST, ALP, and ALT, in addition to reducing the circulating levels of proinflammatory cytokines in intoxicated mice [62]. The combined therapy of TQ with swimming exercise elicited beneficial effects and restored all parameters to normal ranges more than each treatment. Regarding the hepatic and renal function markers, the cotherapy was effectual in returning plasma activities of AST, ALT, ALP, urea, and creatinine to normal levels. Our proposed mechanism for improving and restoring normal levels in this study is referred to improve lipid peroxidation of TQ in addition to anti-inflammatory and immunomodulatory effects and to enhance cellular antioxidant defense mechanisms by TQ and exercise.

**Figure 5:**
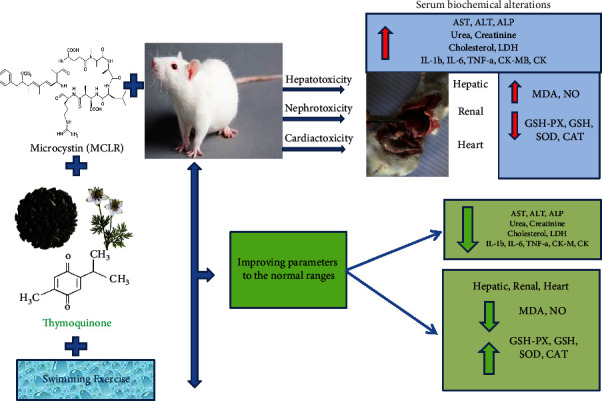
A summary figure for the protective effects of swimming exercise and thymoquinone (5 mg/kg BW) against microcystin-LR(10 *μ*g/kg BW/day).

Treatment with exercise or TQ reduced the MC-induced hepatotoxicity, renal toxicity, and cardiotoxicity, indicated through improved oxidative/antioxidant state and attenuation of cytokines and the biochemical serum parameters (Figure 5).

## 5. Conclusion

The data from this study suggest that MC induces hepatic toxicity, renal toxicity, and cardiotoxicity by elevation of serum hepatic and renal biomarkers in addition to proinflammatory cytokines with the reflection of oxidative state disruption. Treatment with TQ or swimming exercise improved the MC-LR-induced hepatorenal and cardiac injuries in mice; a combination of both treatments showed more improvement than each treatment alone. This enhancement may be explained by improving the tissue's antioxidant defensive mechanisms.

## Figures and Tables

**Table 1:** The biochemical effects of thymoquinone (TQ 5 mg/kg BW once daily for 21 days orally) and water exercise for 30 min daily during microcystin treatment (10 *μ*g/kg BW/day for 14 days) on serum hepatorenal function biomarkers.

	Control	EX Group	TQ Group	MCLR Group	MCLR-EX Group	MCLR-TQ Group	MCLR-TQ-EX Group
AST (U/L)	43.1 ± 4.5(a)	40.2 ± 5.1(a)	40.1 ± 4.8(a)	94.2 ± 9.3(b)	73.2 ± 11.6(c)	55.4 ± 6.8(d)	48.9 ± 2.5(ad)
ALT (U/L)	24.9 ± 2.9(a)	23.7 ± 2.0(a)	23.7 ± 1.9(a)	60.6 ± 7.3(b)	41.2 ± 4.0(c)	39.2 ± 4.0(c)	27.3 ± 5.3(a)
ALP (U/L)	67.6 ± 6.3(a)	62.2 ± 6.6(a)	62.2 ± 6.6(a)	152.3 ± 13.9(b)	106.6 ± 11.5(c)	95.7 ± 10.3(c)	73.4 ± 15.9(a)
Cholesterol (mg/dl)	106.2 ± 9.3(a)	105.0 ± 8.8(a)	100.7 ± 9.5(a)	243.8 ± 22.0(b)	184.1 ± 11.6(c)	164.1 ± 11.6(c)	112.6 ± 13.5(a)
LDH (U/L)	214.8 ± 29.1(a)	211.5 ± 29.6(a)	205.3 ± 29.6(a)	364.6 ± 41.9(b)	286.8 ± 27.6(c)	274.5 ± 27.3(c)	226.3 ± 17.1(a)
CK (U/L)	106.2 ± 14.5(a)	103.4 ± 18.9(a)	101.7 ± 17.0(a)	295.6 ± 20.2(b)	177.7 ± 12.7(c)	164.8 ± 9.5(c)	118.5 ± 13.2(a)
CK-MB (U/L)	35.5 ± 4.5(a)	34.4 ± 6.6(a)	33.9 ± 6.1(a)	146.5 ± 6.8(b)	80.2 ± 6.7(c)	71.3 ± 3.4(d)	40.3 ± 4.9(a)
Urea (mg/dl)	28.6 ± 3.5(a)	27.9 ± 3.3(a)	26.9 ± 3.3(a)	67.4 ± 6.5(b)	48.6 ± 7.5(c)	40.1 ± 7.5(d)	30.9 ± 3.7(a)
Creatinine (mg/dl)	0.3 ± 0.1(a)	0.2 ± 0.1(a)	0.3 ± 0.1(a)	3.6 ± 0.5(b)	1.9 ± 0.2(c)	1.5 ± 0.2(d)	0.5 ± 0.1(a)

Values are presented as means ± SD (*n* = 8), and different superscripts show significant differences (*p* < 0.05) between groups. ALT: alanine transferase; AST: aspartate transferase; ALP: alkaline phosphatase; LDH: lactate dehydrogenase; CK: creatine kinase.

## Data Availability

The data used to support the findings of this study are available from the corresponding author upon reasonable request.

## Abbreviations

ALT: Alanine aminotransferase
ALP: Alkaline phosphatase
AST: Aspartate aminotransferase
CAT: Catalase
GSH: Reduced glutathione
GSH-Px: Glutathione peroxidase
IL-1*β*: Interleukin-1*β*
IL-6: Interleukin-6
LDH: Lactate dehydrogenase
MDA: Malondialdehyde
SOD: Superoxide dismutase
TNF-*α*: Tumor necrosis factor-*α*
TQ: Thymoquinone
MCLR: Microcystin-LR
EX: Exercise.
